# Predicting in‐hospital mortality in patients admitted from the emergency department for pulmonary embolism: Incidence and prognostic value of deep vein thrombosis. A retrospective study

**DOI:** 10.1111/crj.13697

**Published:** 2023-09-19

**Authors:** Teresa Pagano, Irma Sofia Fabbri, Marcello Benedetto, Luca D'Angelo, Giorgio Galizia, Andrea Portoraro, Matteo Guarino, Benedetta Perna, Angelina Passaro, Daniele Cariani, Michele Domenico Spampinato, Roberto De Giorgio

**Affiliations:** ^1^ Department of Translational Medicine University of Ferrara Ferrara Italy; ^2^ School of Emergency Medicine University of Ferrara Ferrara Italy; ^3^ Emergency Medicine Unit, Department of Emergency St. Anna University Hospital Ferrara Italy

**Keywords:** clinical prediction rule, deep vein thrombosis, emergency care, in‐hospital mortality, prognosis, pulmonary embolism, ultrasound

## Abstract

**Background:**

Pulmonary embolism (PE) is one of the most common causes of death from cardiovascular disease. Although deep vein thrombosis (DVT) is the leading cause of PE, its prognostic role is unclear. This study investigated the incidence and prognostic value of DVT in predicting in‐hospital mortality (IHM) in patients admitted from the emergency department (ED) for PE.

**Methods:**

This retrospective cohort study was conducted in the ED of a third‐level university hospital. Patients over 18 years admitted for PE between 1 January 2018 and 31 December 2022 were included.

**Results:**

Five hundred and thirty patients (mean age 73.13 years, 6% IHM) were included. 69.1% of cases had DVT (36.4% unilateral femoral vein, 3.6% bilateral, 39.1% unilateral popliteal vein, 2.8% bilateral, 45.7% distal vein thrombosis and 7.4% iliocaval involvement). Patients who died in hospital had a higher Pulmonary Embolism Severity Index (PESI) (138.6 vs. 99.65, *p* < 0.001), European Society of Cardiology risk class (15.6% vs. 1%, intermediate‐high in 50% vs. 6.4%, *p* < 0.001) and more DVT involving the iliac‐caval vein axis (18.8% vs. 6.6%, *p* = 0.011). PESI class >II, right ventricular dysfunction, increased blood markers of myocardial damage and involvement of the iliocaval venous axis were independent predictors of IHM on multivariate analysis.

**Conclusions:**

Although further studies are needed to confirm the prognostic role of DVT at PE, involvement of the iliocaval venous axis should considered to be a sign of a higher risk of IHM and may be a key factor in prognostic stratification.

## Graphical abstract

In 530 acute PE ED patients (6% in‐hospital death), DVT occurred in 69.1%. While DVT presence did not correlate with death, iliac‐caval involvement emerged as a strong independent predictor (OR 4.82, 95% CI 1.33–17.46). Additionally, PESI class >II (OR 3.2, 95% CI 1.03–9.9), right ventricular dysfunction (OR 5.8, 95% CI 2.18–15.58) and elevated circulatory markers of myocardial injury (OR 4.86, 95% CI 1.51–15.6) played significant roles in short‐term risk stratification.

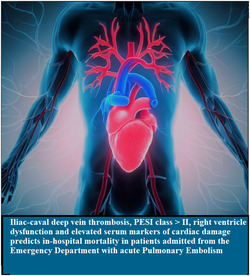

## 1 INTRODUCTION

Deep vein thrombosis (DVT) and pulmonary embolism (PE) are two of the most common diseases of the circulatory system and constitute the definition of venous thromboembolism (VTE).^1^ PE is an acute, recurrent or chronic obstruction of the pulmonary artery vessels caused by the migration of clots (blood, air or fat) from the peripheral venous circulation and is the third most common cardiovascular disease after myocardial infarction and stroke.^2^ Deep venous thrombosis results from the formation of platelet aggregates stabilised by fibrin and primarily affects the venous circulation of the lower limbs. The lower limb circulation includes the proximal venous circulation with the common femoral vein, superficial femoral vein and popliteal vein, and the distal venous circulation with the anterior, posterior tibial and peroneal veins. Traditionally, the three factors of Virchow's triad—venous stasis, hypercoagulability and changes in the endothelial blood vessel lining—contribute to VTE and have been used to explain its development.^3^

Risk factors include pregnancy, prolonged bed rest, surgery, trauma, malignancies, infections, congenital and acquired haemostasis or clotting abnormalities, medications and previous VTE.^2^

Oedema, pain and warmth are non‐specific clinical criteria for the diagnosis of DVT. The differential diagnosis must consider hydrart, contusions or other muscle collections, lymphoedema, venous insufficiency, limb ischaemia, systemic oedema, skin and soft tissue disease, rupture of a popliteal cyst, bed rest, pregnancy, or pill‐induced oedema, and neurological disease.^4^

The diagnosis of DVT is clinical and is made with the aid of ultrasound. It is possible to directly visualise the clot inside the vessel, detect decreased flow in the vein using Doppler analysis or assess altered venous compression using compression ultrasound (CUS), which is the only validated technique for diagnosing DVT. A linear high‐frequency transducer (5–10 MHz) was used to assess DVT, allowing better surface layer exploration. Transverse scanning of the inguinal ligament and popliteal fossa (also called ‘two‐point ultrasound’) is used to perform CUS, and non‐compressible veins were used to find DVT. CUS has many advantages (ease of performance, reproducibility and availability), with >90% sensitivity and >95% specificity in diagnosing thrombosis of the femoral or popliteal veins; however, it is less accurate when the iliac veins or distal district are involved.^5^, ^6^, ^7^

At least 50% of patients with proximal DVT have contextual asymptomatic PE, whereas asymptomatic DVT is found in approximately 80% of patients with TEP. According to the latest guidelines, DVT in the femoropopliteal region poses an increased thromboembolic risk.^2^ Therefore, positive proximal CUS has an increased positive predictive value for PE diagnosis. However, for the distal district, CUS alone is not informative, and further investigations are required. Despite the importance of DVT on PE pathogenesis, data on the prognostic value of DVT are conflicting, especially in the prediction of short‐term mortality after PE diagnosis.

The primary objective of this study was to determine the prognostic value of DVT in predicting in‐hospital mortality (IHM) in patients admitted from emergency department (ED) for PE. The secondary objective was to investigate the incidence and mortality rates in patients at higher risk of DVT.

## 2 MATERIAL AND METHODS

This was a single‐centre, retrospective cohort study conducted between 1 January 2018 and 31 December 2022 in the ED of St. Anne's University Hospital in Ferrara, Italy, a third‐level reference centre for time‐dependent emergencies with >80 000 patients per year. The inclusion criteria were as follows: (i) age >18 years; (ii) a PE diagnosis confirmed by contrast‐enhanced thoracic computed tomography (CT); and (iii) available CUS data.

Exclusion criteria were: (i) concurrent diagnoses at high risk of short‐term death, such as the surgical acute abdomen, cerebral haemorrhage and acute coronary syndrome; (ii) pregnant women; and (iii) inconsistent or incomplete data for European Society of Cardiology (ESC) risk classification.

Patients at higher risk of DVT were defined as follows: (i) patients aged >80 years; (ii) patients with a history of cancer; (iii) patients with recent immobility lasting >4 days or recent surgery <1 month; and (iv) a previous history of thrombosis.

The Pulmonary Embolism Severity Index (PESI) and ESC risk classifications were calculated according to the 2019 ESC Guidelines for the Diagnosis and Treatment of Pulmonary Embolism (2), including vital signs and laboratory data presented during the first evaluation at the ED. The occurrence of in‐hospital deaths was assessed by a single investigator blinded to other clinical data.

The study was conducted in accordance with the Declaration of Helsinki and approved by the local ethics committee. The ‘transparent reporting of a multivariable prediction model for individual prognosis or diagnosis’ statement (TRIPOD statement) was followed in the preparation of manuscript.^8^

### 2.1 Whole‐leg CUS

A dedicated hospital service used GE LOGIQ7 to perform a whole‐leg CUS examination. A linear high‐frequency transducer (5–7.5 MHz) was used to scan the following veins using a standard procedure with the patient in the supine position: common femoral vein, great saphenous vein, distal tract of the great saphenous vein, superficial femoral vein, popliteal vein, posterior tibial vein, peroneal vein, gemellian vein, solean vein, popliteal vein and small saphenous vein.

### 2.2 Statistical analysis

Normally distributed data were described as mean ± standard deviation (SD); non‐normally distributed data were described as the median and interquartile range (IQR); categorical data were expressed as absolute numbers and percentages. Normally distributed data were compared using the *t*‐test for independent samples or Welch's *t*‐test if the variance was unequal between groups. Non‐normally distributed data were compared with the Mann–Whitney U test. The Pearson squared test was used to compare categorical dependent variables between at least two independent groups. Multivariate analysis was performed to test the predictive power of clinical and instrumental data for in‐hospital deaths.

Statistical analyses were performed using SPSS v. 25 (Apache Software Foundation, Chicago, Illinois, USA) and MedCalc version 17.6 (MedCalc Software BVBA).

## 3 RESULTS

**FIGURE 1:**
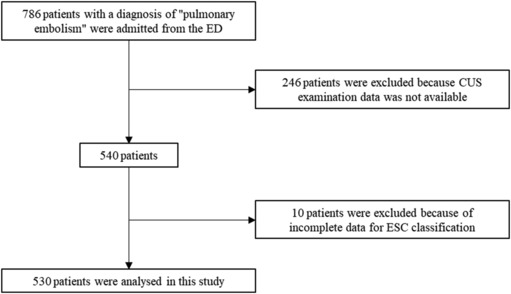
Flow diagram with included and excluded patients.

During the study period, 786 patients with a diagnosis of ‘pulmonary embolism’ were admitted to the hospital from the ED. However, 246 patients were excluded because CUS examination data were not available, 10 patients were excluded because of incomplete data for ESC classification, and 530 patients were analysed in this study (Figure 1). The included patients were male in 41.9% of cases, with a mean age of 73.13 years (standard deviation [SD] = 15.35), a mean PESI of 101.9 (SD = 37.6), an ESC classification risk of ‘high’ in 1.9%, ‘intermediate‐high’ in 9.1%, ‘intermediate‐low’ in 51.7% and ‘low’ in 37.4%, with an IHM rate of 6% (Table 1). Doppler ultrasound examination revealed DVT in 69.1% of cases, unilateral femoral thrombosis in 36.4%, bilateral femoral thrombosis in 3.6%, unilateral popliteal thrombosis in 39.1% and bilateral popliteal thrombosis in 2.8%. Distal thrombosis was found in 45.7% of the cases and iliac‐caval involvement in 7.4% of the cases (Table 2). Compared to uneventful PE patients, IHM patients more often exhibited a decreased state of consciousness (41.2% vs. 13%, *p* < 0.001), lower peripheral oxygen saturation (SpO2) (6.7% vs. 6.47%, *p* = 0.003), lower systolic blood pressure (SBP) (12 vs. 135 mmHg, *p* = 0.014), higher heart rate (HR) (100.24 vs. 89.91 ppm, *p* = 0.008), higher PESI (138.6 vs. 99.65, *p* < 0.001) (Table 3), and more frequently presented with a more severe ESC classification (‘high risk’ at 15.6% vs. 1%, ‘intermediate‐high’ in 50% vs. 6.4%, *p* < 0.001) (Table 4). According to the CUS examination, there was no difference in concurrent DVT rate between patients who died in the hospital and surviving patients and no difference in unilateral, bilateral femoral, popliteal or distal vein involvement; however, patients with in‐hospital death had a significantly higher rate of iliac‐caval involvement (18.8% vs. 6.6%, *p* = 0.011; Table 2).

**TABLE 1:** Characteristics of the population.

Patients features	All patient, *n* = 530	No intra‐hospital death, *n* = 498 (94%)	Intra‐hospital death, *n* = 32 (6%)	*p‐*Value
Age, media (SD), years	73.13 (15.35)	72.28 (15.65)	78.45 (13.98)	0.071
Men, *n* (%)	22 (41.9)	200 (41.3)	13 (43.3)	0.828
History of cancer, *n* (%)	131 (24.7)	116 (23.3)	15 (48.4)	0.02
History of HF, *n* (%)	44 (8.3)	40 (8)	4 (12.5)	0.377
Chronic pulmonary disease, *n* (%)	48 (9.1)	456 (9.3)	2 (6.3)	0.566
Previous DVT, *n* (%)	83 (15.7)	79 (16)	4 (12.9)	0.651
Recent immobility >4 days or recent surgery, *n* (%)	67 (12.6)	60 (12.1)	7 (21.9)	0.1
PESI, media (SD)	101.9 (37.6)	99.65 (36.12)	138.6 (42)	<0.001

Abbreviations: DVT, deep vein thrombosis; HF, heart failure; PESI, Pulmonary Embolism Severity Index; SD, standard deviation.

**TABLE 2:** Doppler ultrasound examination.

	All patient, *n* = 530	No intra‐hospital death, *n* = 498 (94%)	Intra‐hospital death, *n* = 32 (6%)	*p*‐Value
Presence of contextual DVT, *n* (%)	366 (69.1)	343 (70.6)	23 (76.7)	0.476
Unilateral femoral thrombosis, *n* (%)	193 (36.4)	182 (36.5)	11 (34.4)	0.805
Unilateral popliteal thrombosis, *n* (%)	207 (39.1)	196 (39.4)	11 (34.4)	0.575
Bilateral femoral thrombosis, *n* (%)	19 (3.6)	17 (3.4)	2 (6.3)	0.403
Bilateral popliteal thrombosis, *n* (%)	15 (2.8)	14 (2.8)	1 (3.1)	0.917
Distal thrombosis, *n* (%)	242 (45.7)	232 (46.6)	10 (31.3)	0.228
Iliac‐caval involvement, *n* (%)	39 (7.4)	33 (6.6)	6 (18.8)	0.011

Abbreviation: DVT, deep vein thrombosis.

**TABLE 3:** Vital signs and laboratory data presented during the first evaluation.

	All patient, *N* = 530	No intra‐hospital death, *N* = 498 (94%)	Intra‐hospital death, *N* = 32 (6%)	*p‐*Value
Decreased state of consciousness, *n* (%)	75 (14.32)	46 (13)	7 (41.2)	0.001
Respiratory rate (SD), acts per minute	19.5 (5.8)	19.7 (5.9)	17.5 (4.03)	0.13
SpO2, media (SD), %	93.7 (6.5)	93.9 (6.47)	90.2 (6.7)	0.003
SBP, media (SD), mmHg	134.07 (25.43)	135 (25.29)	123 (26.2)	0.014
DBP, media (SD), mmHg	76.03 (13.91)	76 (14.02)	76 (15.07)	0.93
Heart rate (SD), beats per minute	90.54 (20.5)	89.91 (20.02)	100.24 (25.27)	0.008
Shock index, media (SD)	0.7 (0.23)	0.69 (0.22)	0.82 (0.25)	0.04
Body temperature, media (SD),°C	36.6 (2.05)	36.47 (0.70)	38.13 (0.81)	0.25

*Note*: Shock index, calculated as heart rate/systolic blood pressure.

Abbreviations: C, Celsius; DBP, diastolic blood pressure; SBP, systolic blood pressure; SD, standard deviation; SpO2, peripheral oxygen saturation.

**TABLE 4:** European Society of Cardiology classification elements and classes in surviving and deceased patients.

	All patient, *n* = 530	No intra‐hospital death, *n* = 498 (94%)	Intra‐hospital death, *n* = 32 (6%)	*p‐*Value
Hemodynamic instability, *n* (%)	7 (1.3)	5 (1)	2 (6.3)	0.012
PESI class >II, *n* (%)	331 (62.5)	303 (39.2)	28 (87.5)	0.003
Right ventricle dysfunction, *n* (%)	103 (19.4)	81 (22.1)	22 (75.9)	<0.001
Higher circulatory markers of myocardial injury, *n* (%)	176 (33.2)	149 (32)	27 (84.4)	<0.001
ESC classification				
Low risk, *n* (%)	198 (37.4)	195 (39.2)	3 (9.4)	<0.001
Intermediate‐low risk, *n* (%)	274 (51.7)	266 (53.4)	8 (25)	
Intermediate‐high risk, *n* (%)	48 (9.1)	32 (6.4)	16 (50)	
High risk, *n* (%)	10 (1.9)	5 (1)	5 (15.6)	

Abbreviations: ESC, European Society of Cardiology; PESI, Pulmonary Embolism Severity Index.

**FIGURE 2:**
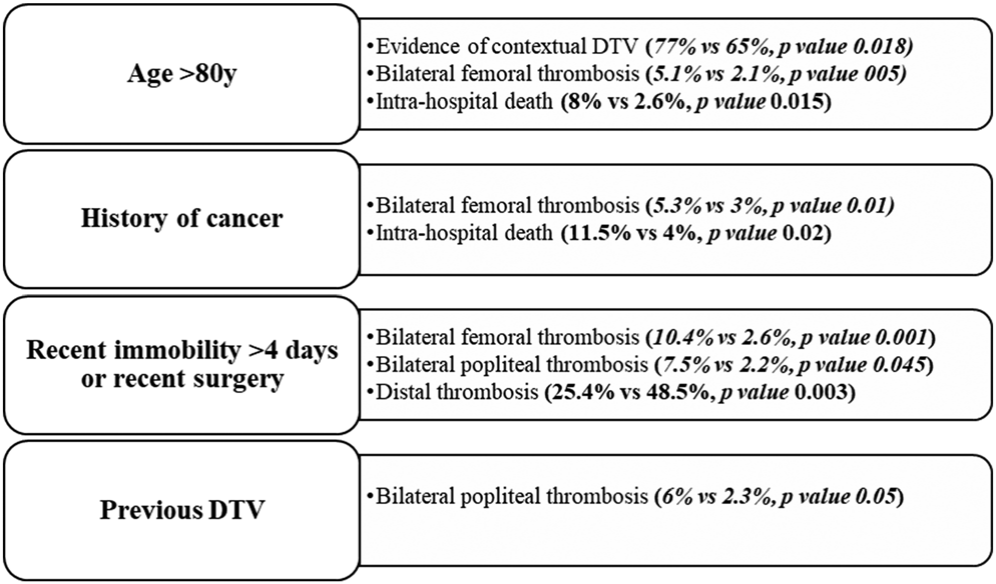
Association between DVT and thrombosis risk factors. *Note*: DVT, deep vein thrombosis; Y, years.

In the evaluation of patients at higher risk of thrombosis, patients aged >80 years more frequently showed positive CUS (77% vs. 65%, *p* = 0.018) and bilateral femoral vein involvement (5.1% vs. 2.6%, *p* = 0.05), with higher IHM (8% vs. 2.6%, *p* = 0.015); patients with a history of cancer were more likely to have bilateral femoral vein involvement (5.3% vs. 3%, *p* = 0.01) and higher IHM (11.5% vs. 4%, *p* = 0.02); patients with recent immobility or recent surgery were more likely to have bilateral femoral vein involvement (10.4% vs. 2.6%, *p* = 0.001), bilateral popliteal involvement (7.5% vs. 2.2%, *p* = 0.0045) and less likely to have distal DVT (25.4% vs. 48.5%, *p* = 0.003); patients with a history of DVT more often had bilateral popliteal DVT (6% vs. 2.3%, *p* = 0.05) (Figure 2).

In multivariate regression analysis, which included the element of ESC classification, significant independent predictors were PESI class >II (odds ratio [OR] 3.2 [95% CI 1.03–9.9]; *p* = 0.044); right ventricular (RV) dysfunction (OR 5.8 [95% CI 2.18–15.58]; *p* = 0.08); higher circulatory markers of myocardial injury (OR 4.86 [95% CI 1.51–15.6]; *p* = 0.008); and iliac‐caval involvement (OR 4.82 [95% CI 1.33–17.46]; *p* = 0.016) (Table 5).

**TABLE 5:** Multivariate regression analysis.

	OR	95% CI	*p‐*Value
Hemodynamic instability	4.4	0.27–70.46	0.29
PESI class >II	3.2	1.03–9.9	0.044
Right ventricular dysfunction	5.8	2.18–15.58	0.08
Higher circulatory markers of myocardial injury	4.86	1.51–15.6	0.008
Iliac‐caval involvement	4.82	1.33–17.46	0.016

Abbreviations: CI, confidence index; OR, odds ratio; PESI, Pulmonary Embolism Severity Index.

## 4 DISCUSSION

Correct prognostic stratification of a patient allows the emergency physician to determine the most appropriate treatment setting according to the risk of IHM, and is, therefore, one of the most important components of the diagnostic‐therapeutic process. Despite the acknowledged role of DVT in PE, with most emboli arising from the lower extremity proximal veins,^9^, ^10^, ^11^ the prognostic role of DVT in short‐term mortality in PE is controversial. The recent 2019 ESC guidelines do not consider thrombotic presence and extension as an essential element for IHM risk stratification, and not even the Hestia criteria for the selection of patients at low risk of acute mortality include DVT among the criteria.^2^ This study showed that DVT was detectable in approximately 70% of patients with PE and the presence of prothrombotic factors such as age >80 years, history of cancer, recent surgery or immobilisation >4 days, and previous DVT was more frequently associated with proximal thrombosis of the lower limbs (see Figure 2). In line with previous studies, patients who underwent in‐hospital death were older, more frequently with cancer, and with higher PESI and ESC class, with hemodynamic instability, PESI class >II, right ventricle dysfunction and higher serum circulating markers of cardiac damage all independent predictors of in‐hospital death.^2^, ^12^, ^13^ Moreover, despite the presence of contextual DVT that did not affect IHM as well as the presence of femoral, popliteal or distal DVT, evidence of iliac‐caval thrombosis was demonstrated to be a strong independent prognostic factor for IHM. In 2010, Jiménez et al.^14^ conducted a prospective cohort study on the prognostic significance of DVT in patients with PE. According to their results, patients with PE and concomitant DVT had increased all‐cause mortality (adjusted hazard ratio [HR], 2.05; 95% confidence interval [CI], 1.24 to 3.38; *p* = 0.005) and PE‐specific mortality (adjusted HR, 4.25; 95% CI, 1.61 to 11.25; *p* = 0.04) compared with those without concomitant DVT. In 2016, Beccattini et al.^15^ published the results of a systematic review and meta‐analysis including nine studies for a total of 8859 patients, demonstrating that patients with concomitant ultrasound‐detectable DVT had a 1.9‐fold increased risk of short‐term death compared to patients without DVT. Later, Jiménez et al.^16^ showed that in 591 hemodynamically stable patients with PE, concomitant DVT was an independent factor for 30‐day mortality with an OR equal to 2.20 (95% CI 1.10–4.38), along with elevated cardiac troponin I and right ventricle dysfunction. In contrast to previous studies, Lee et al. demonstrated the absence of correlation between presence of DVT and PE mortality risk,^17^ whereas Keller et al. in 2020^18^ published the results of a large nationwide inpatient sample of 346 586 PE patients, demonstrating that PE patients with DVT showed better survival (5.4% vs. 20.2%, *p* < 0.001) and lower adverse in‐hospital event rates (9.7% vs. 27.4%, *p* < 0.001) compared to patients with isolated PE. Despite the absence of a correlation between DVT and in‐hospital death in this study, the location of DVT plays a major role in risk stratification. As shown in Table 4 and according to the 2019 ESC guidelines on PE, prognostic factors such as hemodynamic instability, PESI class, right ventricle dysfunction, serum markers of cardiac damage and ESC classification correlated well with IHM; however, while hemodynamic instability was not an independent predictor of IHM, the presence of iliac‐caval DVT was demonstrated to be a strong independent predictor of in‐hospital death with an OR equal to 4.82 (Table 5).

In 2019, Cordeanu et al.^19^ published the results of an observational, prospective registry enrolling all consecutive patients hospitalised in the Vascular Medicine Unit of Strasbourg University Hospital for acute DVT and/or PE, including 1192 patients who were followed up for at least 3 months. According to their results, the presence of DVT was an independent risk factor for PE severity (OR 2.03, 95% 1.47–2.82), and the proximal location of DVT was significantly correlated with an intermediate/high risk of PE (*p* < 0.01), even though no correlation between DVT and 3‐month mortality was found. However, as demonstrated by Bikdeli et al. in 2020^20^ in patients without PE, the location of DVT has a significant role in short‐term morbidity and mortality. According to their results, patients with distal DVT had a lower risk of 90‐day mortality compared with those with proximal DVT (OR, 0.47; 95% CI, 0.40–0.55) and a lower 1‐year hazard of VTE deterioration (HR, 0.83; 95% CI, 0.69–0.99). A possible explanation for the correlation between DVT location and PE severity was provided by Horii et al. in 2011.^21^ According to their study, patients with proximal DVT are more likely to have PE, and proximal PEs correlate with proximal DVTs; acute occlusion of at least 30%–50% of the total cross‐sectional area of the pulmonary arterial bed is sufficient to cause an acute increase in pulmonary arterial pressure (PAP).^22^ RV failure is considered the leading cause of death in patients with severe PE and is unable to support an acute increase in PAP above 40 mmHg, resulting in decreased venous return to the left ventricle and low cardiac output.^23^ PE induces RV inflammatory injury,^24^ RV ischaemia and infarction,^25^ and respiratory failure due to haemodynamic compromise and death.^26^ Shirkhodaie et al. published in 2021^27^ the results of a single‐centre retrospective study analysing the association of DVT and all‐cause mortality at 30 days or 1 year in 189 PE patients treated with catheter‐directed therapy (CDT). In this particular cohort, no association was found; however, no data were provided on the rate of PE patients who had not undergone CDT and on the relationship between concomitant or proximal DVT and the need for CDT. In contrast with previous studies, Wang et al. in 2020^28^ showed a higher incidence of high‐ and intermediate‐risk PE in patients with isolated distal DVT than in those with proximal DVT and bilateral DVT than in those with unilateral DVT (54.5% vs. 39.9%, *p* = 0.03).

As for any retrospective analysis, our study has several limitations that we would like to recognise. Our study included a broad spectrum of patients admitted to the ED for ‘pulmonary embolism’, so the results of this study may not be applicable to hospitalised patients. Moreover, due to the lack of data regarding the role of DVT in IHM in patients with PE, we did not calculate the formal sample size; hence, the low number of included patients and the low percentage of IHM, although in line with the current literature, could have affected the results of our study. Patients were included only with available data on CUS performed by a dedicated hospital service; thus, patients who underwent in‐hospital death without performing a comprehensive CUS examination were excluded, which may alter our conclusion.

## 5 CONCLUSION

PE is a potentially serious disease with high clinical variability. As demonstrated, PESI and ESC risk classification play a fundamental role in risk stratification; however, the presence of iliac‐caval DVT represents an important, major risk factor for in‐hospital death and should be evaluated for a more precise risk stratification.

## AUTHOR CONTRIBUTIONS

Teresa Pagano, Irma Sofia Fabbri, Michele Domenico Spampinato and Roberto De Giorgio have given substantial contributions to the conception and the design of the manuscript. Marcello Benedetto, Giorgio Galizia, Luca D'Angelo and Andrea Portoraro acquisition of data. Michele Domenico Spampinato, Matteo Guarino and Angelina Passaro to analysis and interpretation of the data. All authors have participated in drafting the manuscript. Daniele Cariani and Roberto De Giorgio revised it critically. All authors read and approved the final version of the manuscript. All authors contributed equally to the manuscript and read and approved the final version of the manuscript.

## CONFLICT OF INTEREST STATEMENT

The authors declare no conflict of interest.

## ETHICS STATEMENT

The study was conducted in accordance with the Declaration of Helsinki and approved by the local ethics committee (CE‐AVEC 305/2023/Oss/AOUFe).

## PATIENT CONSENT STATEMENT

Informed consent was waived due to the study's retrospective nature.

## Data Availability

All data are available on request to the corresponding author.
